# Use of Human Chorionic Gonadotropin (HCG) or HCG-Combined Treatments in Male Infertility: A Systematic Review

**DOI:** 10.7759/cureus.94003

**Published:** 2025-10-07

**Authors:** Haris Shoaib, James Duffy, Kamran Ahmed

**Affiliations:** 1 Orthopaedic Surgery, Royal Bolton Hospital, Bolton, GBR; 2 Obstetrics and Gynaecology, Barts Health NHS Trust, London, GBR; 3 Urology, King's College Hospital NHS Foundation Trust, London, GBR

**Keywords:** hcg, human chorionic gonadotropin, male infertility, meta-analysis, randomised controlled trial, systematic review

## Abstract

There exists a lack of consensus on the value of human chorionic gonadotropin (HCG) or HCG-combined therapies for the treatment of male infertility. This article aims to evaluate the efficacy and safety of HCG or HCG-combined interventions in the treatment of male infertility, including men with oligospermia, men with varicocele, men with severe testiculopathy, and men with hypogonadotropic hypogonadism.

We searched the databases Embase, MEDLINE and the Cochrane Central Register of Controlled Trials (CENTRAL), from inception to December 2019. Our selection criteria included randomised controlled trials evaluating the use of HCG or HCG-combined interventions for the treatment of male infertility. We included seven trials, reporting data from 926 men. Study characteristics and methodological assessment were reported for each trial. Estimates of summaries were reported as odds ratios (ORs), with random-effects methods used for calculation. When statistical analyses could not be performed, results were reported narratively.

In our results, HCG showed statistically significant differences in sperm morphology and pregnancy rate (OR 34.49; 95% confidence interval (CI) 1.88-632.45; P=0.02) in men with varicocele who were treated for three months following varicocelectomy. The use of HCG-combined therapy for three months was associated with increased pregnancy rates in men with oligospermia (OR 2.91; 95% CI 1.57-5.38; P=0.0007), but no statistically significant differences were observed in men with severe testiculopathy (OR 5.13; 95% CI 0.27-98.56; P=0.28). There was no reporting of pregnancy rate for men with hypogonadotropic hypogonadism. There was absent or limited reporting of secondary outcomes, including live births, birth weight, gestational age at delivery and adverse events.

In conclusion, there is limited evidence to support the use of HCG and a lack of compelling evidence to suggest HCG-combined interventions for the treatment of male infertility. There is a need for more research to be carried out.

## Introduction and background

An unfortunate reality for a substantial number of couples is the inability to conceive children. It is estimated that within 12 months of unprotected sexual intercourse, 15% of couples are unable to achieve pregnancy and therefore resort to seeking medical treatment [1]. Infertility due to male factors alone may be found in approximately 40% of all cases [2] and can be attributed to hormonal, genetic or anatomical causes. These causes can sometimes be irreversible, but in a significant number of cases (40-50%), there is no identifiable cause [3].

Whilst a significant proportion of male factor infertility is due to idiopathic infertility, there are many known causes of male infertility for which there are pharmacological first-line treatments. Gonadotropin therapy is widely used for the improvement of spermatogenesis in males with impaired pituitary or hypothalamic function [4,5], but studies have shown that it can also be used for the same purpose in the treatment of idiopathic male factor infertility [6]. Gonadotropin therapy consists of the administration of gonadotropins such as human chorionic gonadotropin (HCG), human menopausal gonadotropin (HMG) or follicle-stimulating hormone (FSH), among others, where HCG can be delivered alone or in combination with other gonadotropins. HCG is an analogue of luteinizing hormone (LH), and so the purpose of HCG administration is to stimulate Leydig cell function, thereby increasing intra-testicular testosterone levels.

In the treatment of male infertility, the use of HCG or HCG-combined interventions is commonly confined to the treatment of hypogonadotropic hypogonadism (HH). As patients with this disorder experience suppressed levels of gonadotropins such as FSH, LH or testosterone, HCG or HCG-combined interventions are effective in increasing these levels and re-initiating spermatogenesis [7,8]. Whilst HCG is mainly used in the treatment of HH, a few studies have shown the effectiveness of prolonged use of HCG alone [9,10] or HCG-combined interventions in the treatment of male factor infertility with multiple aetiologies [11].

However, despite these studies, there is a lack of consensus regarding the use and value of HCG in the treatment of multiple male infertility aetiologies. There is a considerable psychosocial and economic burden to male infertility, with clear links to marital strain, reduction in quality of life and influence on mental health. Recently updated national guidelines further encourage the need for improved, evidence-based therapies to manage gaps in consensus regarding HCG and HCG-combined treatment options [12,13]. Therefore, the purpose of this systematic review is to evaluate the efficacy and safety of HCG or HCG-combined interventions in the treatment of male infertility, including men with oligospermia, varicocele, severe testiculopathy, and HH.

## Review

Methods

We designed a protocol with clearly defined objectives containing study selection criteria, defined approaches for assessment of study quality and outcomes, and statistical methodology. An extensive literature search was carried out, searching: (1) MEDLINE, (2) Embase and (3) Cochrane Central Register of Controlled Trials (CENTRAL) databases from inception to December 2019. The following Medical Subject Headings (MeSH) terms were used to search the register: (1) HCG; (2) male infertility; and (3) treatment (see Appendix A). Filters were applied to each database to search for randomised controlled trials only.

One review author (H.S.) independently screened titles and abstracts. The full texts of the selected studies were then critically reviewed for eligibility. We included randomised controlled trials of infertile men receiving HCG or HCG-combined interventions for their treatment. Any non-randomised studies were excluded. Exclusion criteria also included animal studies, non-male populations, interventions not involving HCG, or trials without fertility-related outcomes. One review author (H.S.) was involved in the independent extraction of data using a data extraction form. Characteristics of each study were extracted, including study design, setting, number of participants, population, interventions and outcomes. All relevant raw data were extracted from every study, and the methodological quality of each study was independently assessed using the Jadad criteria by one review author (H.S.) with discrepancies resolved via consultation with co-authors [14].

The primary outcomes include World Health Organisation (WHO) sperm parameters: (1) sperm concentration, (2) sperm motility and (3) sperm morphology; and the secondary outcomes include: (1) pregnancy, (2) method of confirmation, (3) type of pregnancy, (4) live births, (5) gestational age at delivery, (6) birth weight, (7) neonatal mortality, (8) major congenital abnormalities and (9) adverse events. If additional data were required or any data were missing, we contacted the relevant authors to submit requests for the data. Any discrepancies were resolved through conversation between the reviewers or by contacting the relevant authors.

For the analysis of results, we used Revision Manager 5.3 (The Cochrane Collaboration, London, UK). We used random-effects (Mantel-Haenszel) methods for the calculation of summary estimates. We present the summary effect size as an odds ratio (OR) with 95% confidence intervals (CIs). I^2^ statistics were used for the assessment of between-study heterogeneity. We aimed to present summary effects for the WHO sperm parameters as weighted mean differences with random-effects calculations; however were unable to do so due to limitations in the data.

Results

Study Selection

**Figure 1:**
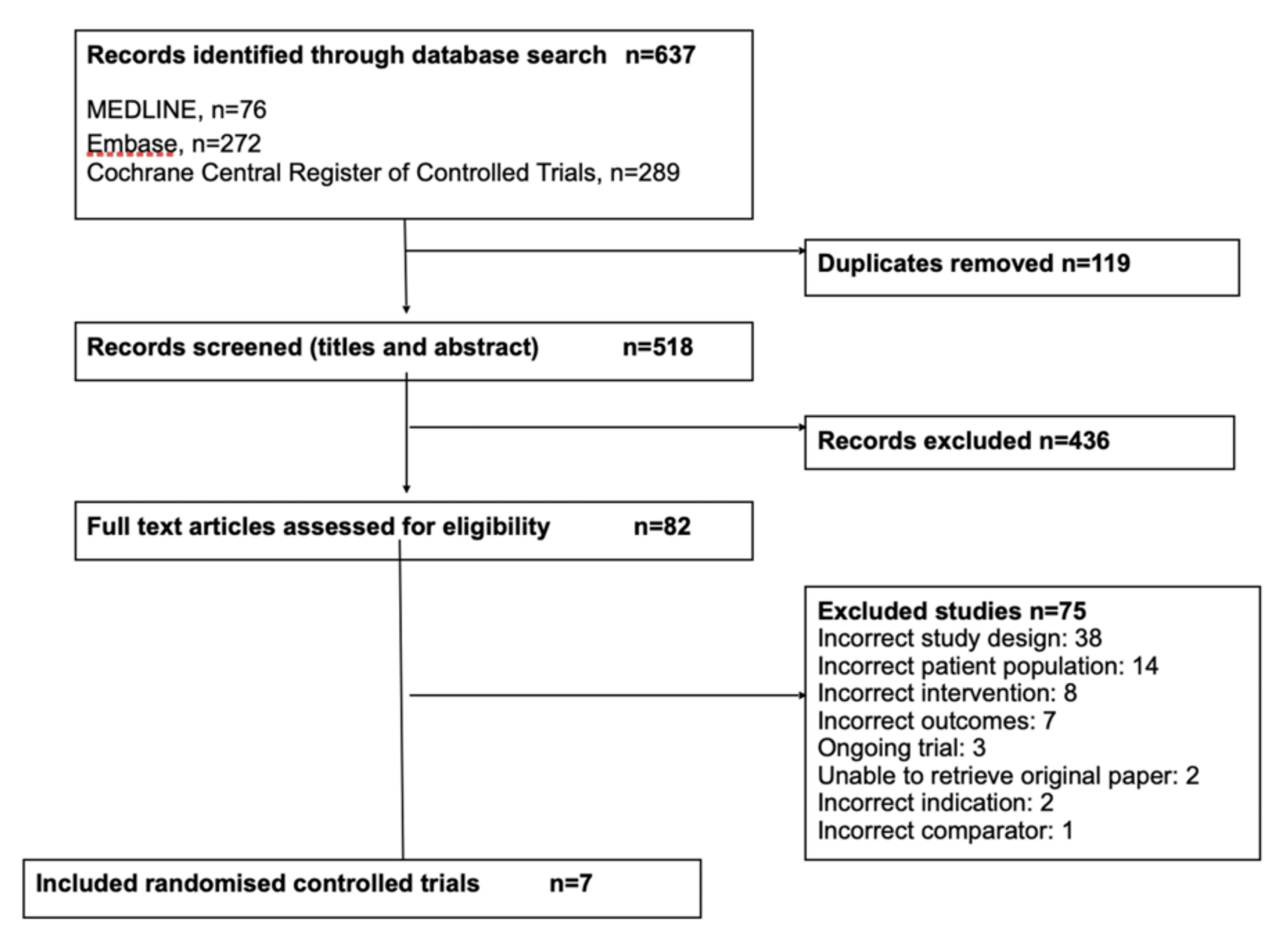
Flow diagram of included studies.

We identified 637 records, excluding 119 duplicate records. Title and abstract screening narrowed records down to 82 studies to be assessed for eligibility (Figure 1). After full-text screening, seven randomised controlled trials met the inclusion criteria, with data reported from 926 men [6,15-20]. Three trials were ongoing.

*Study Characteristics* The included randomised trials evaluated 12 contrasting interventions (Table 1). The interventions covered the use of HCG alone [6,16] or in combination with other interventions such as HMG [17,19], clomiphene [14], recombinant human FSH (rhFSH) [15], gonadotropin-releasing hormone agonist (GnRH-a) and FSH (20). The use of HCG alone was also compared with other interventions such as testosterone [18], rhFSH and HMG [6]. The authors’ assessments of the methodological quality of included studies are detailed in Table 1.

**Table 1:** Characteristics of included randomised trials. * Amirzargar 2012 multi-arm trial comparing HCG, HMG, rhFSH, and no treatment [6]. ¶ Habous 2018 multi-arm trial comparing HCG, clomiphene and clomiphene in combination with HCG [16]. # La Vigneria 2016 multi-arm trial comparing HCG, testosterone-gel formulation, testosterone-undecanoate and testosterone-ethanoate [18]. GnRH-a: gonadotropin-releasing hormone agonist; HCG: human chorionic gonadotropin; HMG: human menopausal gonadotropin; IU: international unit; od: once daily; mg: milligrams; rhFSH: recombinant human follicle-stimulating hormone

Study	Country	n	Intervention 1	Intervention 2	Jadad Scale
HCG 2000 IU and HMG compared with placebo in men with oligospermia					
Zhao 2019 [19]	China	316	HCG 2000 IU twice a week + HMG 150 IU thrice a week	Placebo	4
HCG 2500 IU and HMG compared with placebo in men with oligospermia					
Knuth 1987 [17]	Germany	37	HCG 2500 IU twice a week + HMG 150 IU thrice a week	Placebo	4
HCG, GnRH-a, and rhFSH with no treatment in men with severe testiculopathy					
Foresta 2009 [20]	Italy	87	HCG 2000 IU twice a week + GnRH-a 2.75mg monthly + rhFSH 150 IU every other day	No treatment	3
HCG compared with no treatment following varicocelectomy					
Amirzargar 2012* [6]	Iran	60	HCG 5000 IU weekly	No treatment	2
HCG compared with HMG following varicocelectomy					
Amirzargar 2012* [6]	Iran	46	HCG 5000 IU weekly	HMG 75 IU thrice a week	2
HCG compared with rhFSH following varicocelectomy					
Amirzargar 2012* [6]	Iran	57	HCG 5000 IU weekly	rhFSH 75 IU thrice a week	2
HCG and rhFSH 150 IU compared with HCG and rhFSH 225 IU in men with hypogonadotropic hypogonadism					
Bouloux 2013 [15]	Australia Denmark Germany United Kingdom	30	HCG 1500 IU twice a week + rhFSH 150 IU twice a week	HCG 1500 IU twice a week + rhFSH 225 IU thrice a week	2
HCG compared with clomiphene in men with hypogonadotropic hypogonadism					
Habous 2018¶ [16]	Saudi Arabia	168	HCG 5000 IU twice a week	Clomiphene 50mg od	3
HCG and clomiphene compared with clomiphene in men with hypogonadotropic hypogonadism					
Habous 2018¶ [16]	Saudi Arabia	154	HCG 5000 IU twice a week + Clomiphene 50mg od	Clomiphene 50mg od	3
HCG compared with testosterone gel in men with hypogonadotropic hypogonadism					
La Vigneria 2016# [18]	Italy	20	HCG 2000 IU twice a week	Testosterone gel 60mg every four hours	1
HCG compared with testosterone undecanoate in men with hypogonadotropic hypogonadism					
La Vigneria 2016# [18]	Italy	20	HCG 2000 IU twice a week	Testosterone undecanoate 1000mg every three months	1
HCG compared with testosterone ethanoate in men with hypogonadotropic hypogonadism					
La Vigneria 2016# [18]	Italy	20	HCG 2000 IU twice a week	Testosterone ethanoate 250mg monthly	1

*Study Outcomes* The randomised trials varied in size, ranging from 30 men [15] to 316 men [19] and were conducted in high-income [15-18,20] and middle-income settings [6,19]. No trials were conducted in low-income countries. Two randomised trials reported data from 353 men with oligospermia [17,19], one trial reported data from 87 men with severe testiculopathy [20], one trial reported data from 113 men with varicocelectomy [6], and three trials reported data from 352 men with hypogonadotroic hypogonadism [15,16,18]. Four trials measured semen characteristics and pregnancies [6,17,19,20]. Two studies measured only semen characteristics [13,16]. Habous et al. reported no relevant clinical outcomes [16]. The outcomes reported by each trial are recorded in Table 2.

**Table 2:** Outcomes reported by studies. + denotes that a reported outcome was extracted; - denotes that an outcome was not reported; * denotes that an outcome was reported but not extracted due to statistical reporting methods unsuitable for meta-analysis (e.g., median/range reporting) GnRH-a: gonadotropin-releasing hormone agonist; HCG: human chorionic gonadotropin; HMG: human menopausal gonadotropin; IU: international unit; od: once daily; mg: milligrams; rhFSH: recombinant human follicle-stimulating hormone

Study	Pregnancy	COMMIT outcomes							WHO sperm parameters			Adverse events
		Method of confirmation	Type of pregnancy	Live births	Gestational age at delivery	Birth weight	Neonatal mortality	Major congenital abnormalities	Sperm concentration	Sperm motility	Sperm morphology	
HCG 2000 IU and HMG compared with placebo in men with oligospermia												
Zhao 2019 [19]	+	+	-	-	-	-	-	-	*	*	*	-
HCG 2500 IU and HMG compared with placebo in men with oligospermia												
Knuth 1987 [17]	+	-	-	-	-	-	-	-	*	*	*	+
HCG, GnRH-a, and rhFSH with no treatment in men with severe testiculopathy												
Foresta 2009 [20]	+	+	-	-	-	-	-	-	*	*	*	-
HCG compared with no treatment following varicocelectomy												
Amirzargar 2012 [6]	+	-	-	-	-	-	-	-	*	*	*	+
HCG compared with HMG following varicocelectomy												
Amirzargar 2012 [6]	+	-	-	-	-	-	-	-	*	*	*	+
HCG compared with rhFSH following varicocelectomy												
Amirzargar 2012 [6]	+	-	-	-	-	-	-	-	*	*	*	+
HCG and rhFSH 150 IU compared with HCG and rhFSH 225 IU in men with hypogonadotropic hypogonadism												
Bouloux 2013 [15]	-	-	-	-	-	-	-	-	*	*	*	+
HCG compared with clomiphene in men with hypogonadotropic hypogonadism												
Habous 2018 [16]	-	-	-	-	-	-	-	-	-	-	-	-
HCG and clomiphene compared with clomiphene in men with hypogonadotropic hypogonadism												
Habous 2018 [16]	-	-	-	-	-	-	-	-	-	-	-	-
HCG compared with testosterone gel in men with hypogonadotropic hypogonadism												
La Vigneria 2016 [18]	-	-	-	-	-	-	-	-	-	*	*	-
HCG compared with testosterone undecanoate in men with hypogonadotropic hypogonadism												
La Vigneria 2016 [18]	-	-	-	-	-	-	-	-	-	*	*	-
HCG compared with testosterone ethanoate in men with hypogonadotropic hypogonadism												
La Vigneria 2016 [18]	-	-	-	-	-	-	-	-	-	*	*	-

For the six included randomised trials that reported relevant clinical outcomes, there was a lack of reported data on the primary outcomes in each trial that would allow for the statistical calculations of mean differences and 95% CIs [6,15,17-20]. Therefore, in order to make comparisons of the primary outcomes, the WHO sperm parameters, the authors used P-values from the original publications. Statistical calculations, however, have been carried out by the authors for the secondary outcome, pregnancy rate, and these have been reported as ORs with 95% CIs and P-values.

HCG Compared With No Treatment

Amirzargar et al. compared 25 men with varicocele receiving HCG treatment after varicocelectomy and 35 men with varicocele receiving no treatment after varicocelectomy [6]. The trial measured WHO sperm parameters 8 to 10 weeks after completion of monthly treatment periods, with treatment lasting three months [6]. Pregnancies were recorded up to six months after the end of treatment. All sperm parameters for both treatment groups were compared with pre-treatment sperm characteristics [6]. The comparison of sperm concentration of both the HCG treatment and no treatment groups with pre-treatment characteristics did not indicate any statistical significance (P=0.803 and P=0.157, respectively), nor did the comparison of sperm motility (P=0.582 and P=0.358, respectively) [6]. However, both groups showed statistical significance in the percentage of normal sperm morphology greater than 30% after treatment (P=0.007 and P=0.038, respectively) [6]. There is also evidence to show increased pregnancy rates in men following varicocelectomy when comparing HCG treatment with no treatment (OR 34.49; 95% CI 1.88-632.45; P=0.02), indicating statistical significance. Limited adverse events were reported.

HCG Compared With Other Treatments

Two trials, reporting data from 118 men, compared the use of HCG with alternative treatments for male infertility [6,18]. Amirzargar et al. compared 25 men with varicocele receiving HCG treatment for three months after varicocelectomy and 21 men with varicocele receiving HMG treatment for three months after varicocelectomy [6]. Sperm parameters for both interventions were compared with pre-treatment sperm characteristics [6]. Comparison of sperm concentration after treatment did not indicate statistical significance in either the HCG or HMG treatment groups (P=0.803 and P=0.130, respectively). Sperm motility comparison between the groups (P=0.582 and P=0.023, respectively) showed statistical significance in the HMG group, and when comparing sperm morphology with pre-treatment characteristics, both groups indicated statistical significance (P=0.007 and P=0.014, respectively). Comparison of pregnancy rate between the two treatment groups (OR 0.35; 95% CI 0.11-1.18; P=0.09) did not show any statistically significant changes.

Amirzargar et al. also compared the same human chorionic treatment group with 32 men receiving rhFSH treatment after varicocelectomy [6]. In the HCG treatment group, comparison of sperm concentration (P=0.803) and sperm motility (P=0.130) with pre-treatment characteristics did not indicate any statistical significance. However, the comparison of sperm motility did show a statistically significant difference (P=0.007). For the rhFSH treatment group, comparison of sperm concentration (P=0.027), sperm motility (P=0.027) and sperm morphology (P=0.015) with pre-treatment sperm characteristics all indicated statistically significant differences. Statistical significance was also indicated in the comparison of pregnancy rate between the two treatment groups (OR 0.28; 95% CI 0.09-0.85; P=0.02).

La Vigneria et al. compared the use of HCG with three different testosterone formulations: testosterone gel, testosterone undecanoate and testosterone ethanoate [16]. Forty men with HH were divided equally into the four groups and were treated for six months. La Vigneria et al. measured sperm motility and morphology at the end of a six-month treatment period [18]. P-values from the publication were used for comparisons. The trial showed a decrease in progressive motility, as a change from baseline, in all three testosterone formulations when compared with the HCG treatment group (P<0.05), indicating statistical significance [18]. There were no differences observed in normal sperm morphology [16]. No adverse events were reported.

HCG-Combined Treatment Compared With No Treatment

HCG and HMG: Knuth et al. compared 17 men with oligospermia receiving 2500 International Units (IU) HCG and HMG with 20 men with oligospermia receiving placebo treatment [17]. Knuth et al. measured WHO sperm parameters every month for four months in a follow-up cycle, after a 13-week treatment period [17]. Pregnancies were recorded up to six months after the end of treatment. Sperm parameters in both treatment groups were compared with pre-trial semen characteristics. Combined HCG and HMG treatment showed no statistically significant differences in sperm concentration, with the men receiving placebo treatment experiencing higher mean sperm concentrations during the first (P=0.16) and second (P=0.05) months, despite similar mean results at pre-trial examinations [17]. In the group receiving placebo treatment, a higher percentage of normal sperm morphology was reported in the first (P=0.09), second (P=0.10) and third months (P=0.64) of treatment. However, these results were not statistically significant. Both combined gonadotropin treatment and placebo treatment showed no effect on sperm motility [17]. The comparison of pregnancy rates between the groups (OR 6.61; 95% CI 0.30-147.85; P=0.23) did not indicate statistical significance. Knuth et al. reported febrile illness as an adverse event for two men [17].

Zhao et al. compared 158 men with oligospermia receiving 2000 IU HCG and HMG with 158 men with oligospermia receiving placebo treatment [19]. Zhao et al. measured WHO sperm parameters every month in a three-month treatment period and measured pregnancies three months after the end of treatment [19]. Zhao et al. categorised the men into three groups: lower, medium and higher levels, dependent upon participants’ plasma concentrations of inhibin B [19]. During the first two months of treatment, no statistically significant differences were observed in any of the seminal parameters across all three groups (P>0.05) [19]. However, in the third month of treatment, statistically significant differences were reported in sperm concentration, motility and morphology in the medium and higher level groups for combined gonadotropin therapy (P<0.05) [19]. Pregnancies were confirmed by ultrasound scan [19] and comparison of pregnancy rates between the treatment and placebo group (OR 2.91; 95% CI 1.57-5.38; P=0.0007) indicated statistical significance. No adverse events were reported.

HCG, GnRH-a and rhFSH: Foresta et al. compared 57 men with severe testiculopathy receiving a treatment combination of HCG, GnRH-a and rhFSH with 30 men with severe testiculopathy receiving no treatment [20]. Foresta et al. measured WHO sperm parameters every month in a four-month treatment period and measured pregnancies three months after the end of treatment [20]. During the first month of treatment with just GnRH-a, no notable differences were observed in any of the seminal parameters [20]. However, after three further months of combined gonadotropin treatment, statistically significant differences were reported in sperm count and sperm morphology (P<0.05) [20]. No statistically significant differences were observed in sperm motility [20]. Pregnancies were confirmed by the measurement of female partners’ β-HCG plasma levels [20], and comparison of pregnancy rate between the two intervention groups (OR 5.13; 95% CI 0.27-98.56; P=0.28) did not indicate any statistical significance. No adverse events were reported.

HCG-combined treatment compared with other treatments: Bouloux et al. compared 15 men with HH receiving a combination of HCG and 150 IU rhFSH with 15 men with HH receiving HCG and 225 IU rhFSH [15]. Bouloux et al. measured WHO sperm parameters every six weeks in a 12-month treatment period [15]. Comparison of sperm concentrations was not statistically significant (P>0.05), with 33% of men in each group remaining completely azoospermic after treatment [15]. At the end of the combined treatment for both groups, the overall mean fraction of progressive sperm cells and morphologically normal sperm cells was 35% and 24% respectively [15]. Thirteen adverse events were reported, including hyperglycaemia, gynaecomastia, pilonidal cysts, haemorrhoids and acne [15].

Discussion

There is limited evidence to support the use of HCG in the treatment of male infertility, with conflicting evidence on its use in combination with other gonadotropins. There were a few statistically significant differences in sperm parameters, including sperm concentration and sperm motility, across all interventions. Sperm morphology and pregnancy rate indicated more statistically significant differences, though these were not conclusive. Comparison of HCG treatment with alternative treatments has not been sufficiently evaluated; however, the limited evidence is conflicting, and, therefore, we are unable to understand the true treatment effect. In the consideration of safety outcomes, there was limited reporting of adverse events and no reporting of patient-reported outcomes for all interventions.

The strengths of this systematic review include a well-defined search strategy, comprehensive methodological assessment, and narrative synthesis. The primary outcome, sperm parameters, was reported by six out of seven included studies. A wider range of outcomes, including COMMIT outcomes [21] and adverse events, enabled more effective evaluation of the varying infertility treatments. Large variation in settings, patient populations and treatment interventions should enable this review to be applied to a broader clinical context within our speciality.

However, systematic reviews do not come without their limitations. The use of a single reviewer in the screening of studies means studies may have been missed, without a second reviewer to resolve disputes, which could have resulted in significant changes in the findings of this review. Whilst the search strategy was able to identify seven randomised controlled trials, the pool of participants was relatively low. The majority of included randomised trials recruited fewer than 100 men and so would maintain a deficiency in the demonstration of differences between HCG therapy and alternative treatments.

Included studies, which observed similar outcomes and treatments, showed heterogeneity in the reporting of treatment data, with some studies reporting mean data in monthly intervals and others reporting mean values at the end of treatment only, making comparisons of intervention effects more difficult. There was limited reporting of usable data, as there was significant heterogeneity in primary outcome measures and a lack of relevant reported data in the randomised trials. Therefore, when considering meta-analysis, we were unable to perform statistical analyses for any primary outcomes, rendering the authors unable to make meaningful comparisons. There was reporting of only two secondary outcomes, the method of pregnancy confirmation and pregnancy rate, for which statistical analysis was carried out. There was limited reporting of adverse events, including headache, acne, febrile illness and nausea.

As evidence of all comparisons was limited, any conclusions regarding the use of HCG in the treatment in the treatment of infertility should be made with caution. This systematic review highlights the lack of high-quality, credible evidence to offer strong support for the use of HCG in male infertility treatment. HCG as a treatment has been shown to be effective in men with HH, where it is most commonly applied [22]. However, in the treatment of idiopathic male infertility, uncertainty lies over its value and evidence from research has currently led to a lack of consensus on the ideal treatments for male factor infertility, despite expert guidelines.

Taking into consideration the evidence available, use of HCG alone for the induction of spermatogenesis in infertile men has been shown to be effective, more specifically for patients with varicocele or hypogonadism, in line with conclusions offered by Khourdaji et al. and Ring et al. [3,22]. However, in men with idiopathic infertility, studies suggest limited evidence for effective treatment [17,23]. Whilst there is an absence of consensus on the use of HCG alone in the treatment of male infertility, recent studies have shown its benefit in combination with other gonadotropins or treatments in improving outcomes such as sperm parameters [24]. Another recent review highlighted that the use of combined gonadotropin therapy may be effective in restoring fertility in patients at a late stage of insufficiently treated disease, in addition to already established adequate treatment [25], suggesting that combined gonadotropin therapy may be effective in combination with alternative therapies for infertility treatment; however further research is required into the use and practicality of combined therapies.

Although there is some evidence to support the use of HCG treatment, consideration of clinical factors is imperative in ensuring its appropriate management. Gonadotropin therapy has been reported to be of great expense as well as invasive in its nature of delivery [3], factors that can influence its choice as a treatment option. It is also important to ensure patients have adequate support in the management of their infertility. A need for awareness of infertility risk factors is imperative to ensure fertility preservation [26]. The use of HCG or combined gonadotropin therapy requires commitment to treatment, despite knowledge of a chance of failed therapy dependent upon pre-therapy conditions [27]. The acknowledgement of potential psychosocial issues related to gonadotropin therapy is paramount in its appropriate delivery.

Clinically, HCG-based interventions remain particularly relevant in HH but may also offer adjunctive use in patients following varicocelectomy. Cost-effectiveness must be considered as gonadotropin therapy can be expensive and resource-intensive, which limits its access. Patient counselling should include discussions regarding treatment burden and psychosocial impacts, with emerging evidence suggesting roles for tailored therapy and precision medicine [12,13].

Whilst there is evidence to support the effectiveness of HCG in the induction of spermatogenesis, further research is required to evaluate its efficacy in the maintenance of spermatogenesis. A greater number of prospective studies are required to explore historical gonadotropin therapy and evaluate its effectiveness in a wider range of infertile male populations, including idiopathic male infertility. In addition to this, human chorionic treatment needs to be trialled for longer treatment periods to evaluate long-term use of the treatment, its effect on gonadotropin deficiency and also its ability to stabilise sperm parameters.

## Conclusions

There is an absence of consensus on the use of HCG or HCG combined therapies for the treatment of male infertility. Current evidence suggests that HCG therapy can be effective for the treatment of infertility disorders such as varicocele or hypogonadism. However, there is limited evidence reported in this review for the efficacy of HCG or combined gonadotropin therapy on other male infertility populations, such as idiopathic male infertility. Therefore, there is a need for more research in larger-scale populations and multi-centred settings, in order to provide more evidence on the evaluation of HCG or combined gonadotropin therapies for the treatment of male infertility.

## Appendices

Appendix A

Search Strategy and Figures

Search from: Database inception to 23/12/2019

Search: Chorionic Gonadotropin ADJ3 Human OR HCG OR Human Chorionic ADJ3 Gonadotropin AND infertility, male OR subfertility, male AND treatment* OR Therap* 

PUBMED 

Results = 77 (Limit articles to - Randomised Controlled Trials, Humans, English Language)

CENTRAL 

Results = 289 (77 duplicates)

EMBASE

Results = 272 (51 duplicates) (Limit articles to - Randomised Controlled Trials)

Total searches - 637

Total duplicates - 119

Total screened from databases - 518

## References

[1] Jungwirth A, Giwercman A, Tournaye H, Diemer T, Kopa Z, Dohle G, Krausz C (2012). European Association of Urology guidelines on male infertility: the 2012 update. Eur Urol.

[2] Jodar M, Soler-Ventura A, Oliva R (2017). Semen proteomics and male infertility. J Proteomics.

[3] Ring JD, Lwin AA, Köhler TS (2016). Current medical management of endocrine-related male infertility. Asian J Androl.

[4] Whitcomb RW, Crowley WF Jr (1990). Clinical review 4: diagnosis and treatment of isolated gonadotropin-releasing hormone deficiency in men. J Clin Endocrinol Metab.

[5] Zitzmann M, Nieschlag E (2000). Hormone substitution in male hypogonadism. Mol Cell Endocrinol.

[6] Amirzargar MA, Yavangi M, Basiri A (2012). Comparison of recombinant human follicle stimulating hormone (rhFSH), human chorionic gonadotropin (HCG) and human menopausal gonadotropin (HMG) on semen parameters after varicocelectomy: a randomized clinical trial. Iran J Reprod Med.

[7] Kim ED, Crosnoe L, Bar-Chama N, Khera M, Lipshultz LI (2013). The treatment of hypogonadism in men of reproductive age. Fertil Steril.

[8] Farhat R, Al-zidjali F, Alzahrani AS (2010). Outcome of gonadotropin therapy for male infertility due to hypogonadotrophic hypogonadism. Pituitary.

[9] Burger HG, Baker HW (1984). Therapeutic considerations and results of gonadotropin treatment in male hypogonadotropic hypogonadism. Ann N Y Acad Sci.

[10] Vicari E, Mongioì A, Calogero AE, Moncada ML, Sidoti G, Polosa P, D'Agata R (1992). Therapy with human chorionic gonadotrophin alone induces spermatogenesis in men with isolated hypogonadotrophic hypogonadism - long-term follow-up. Int J Androl.

[11] Check JH (2007). Treatment of male infertility. Clin Exp Obstet Gynecol.

[12] Pozzi E, Corsini C, Salonia A (2025). Medical therapy for male infertility. Curr Opin Urol.

[13] Muir CA, Zhang T, Jayadev V, Conway AJ, Handelsman DJ (2025). Efficacy of gonadotropin treatment for Induction of spermatogenesis in men with pathologic gonadotropin deficiency: a meta-analysis. Clin Endocrinol (Oxf).

[14] Jadad AR, Moore RA, Carroll D, Jenkinson C, Reynolds DJ, Gavaghan DJ, McQuay HJ (1996). Assessing the quality of reports of randomized clinical trials: is blinding necessary?. Control Clin Trials.

[15] Bouloux PM, Nieschlag E, Burger HG (2003). Induction of spermatogenesis by recombinant follicle-stimulating hormone (puregon) in hypogonadotropic azoospermic men who failed to respond to human chorionic gonadotropin alone. J Androl.

[16] Habous M, Giona S, Tealab A (2018). Clomiphene citrate and human chorionic gonadotropin are both effective in restoring testosterone in hypogonadism: a short-course randomized study. BJU Int.

[17] Knuth UA, Hönigl W, Bals-Pratsch M, Schleicher G, Nieschlag E (1987). Treatment of severe oligospermia with human chorionic gonadotropin/human menopausal gonadotropin: a placebo-controlled, double blind trial. J Clin Endocrinol Metab.

[18] La Vignera S, Condorelli RA, Cimino L, Russo GI, Morgia G, Calogero AE (2016). Late-onset hypogonadism: the advantages of treatment with human chorionic gonadotropin rather than testosterone. Aging Male.

[19] Zhao N, Lu XL, Li JT, Zhang JM (2019). Treatment of idiopathic oligozoospermia with combined human chorionic gonadotropin/human menopausal gonadotrophin: a randomised, double-blinded, placebo-controlled clinical study. Andrologia.

[20] Foresta C, Selice R, Moretti A, Pati MA, Carraro M, Engl B, Garolla A (2009). Gonadotropin administration after gonadotropin-releasing-hormone agonist: a therapeutic option in severe testiculopathies. Fertil Steril.

[21] Duffy JM, Bhattacharya S, Curtis C (2018). A protocol developing, disseminating and implementing a core outcome set for infertility. Hum Reprod Open.

[22] Khourdaji I, Lee H, Smith RP (2018). Frontiers in hormone therapy for male infertility. Transl Androl Urol.

[23] Kamischke A, Behre HM, Bergmann M, Simoni M, Schäfer T, Nieschlag E (1998). Recombinant human follicle stimulating hormone for treatment of male idiopathic infertility: a randomized, double-blind, placebo-controlled, clinical trial. Hum Reprod.

[24] Rajkanna J, Tariq S, Oyibo SO (2016). Successful fertility treatment with gonadotrophin therapy for male hypogonadotrophic hypogonadism. Endocrinol Diabetes Metab Case Rep.

[25] Rohayem J, Tüttelmann F, Mallidis C, Nieschlag E, Kliesch S, Zitzmann M (2014). Restoration of fertility by gonadotropin replacement in a man with hypogonadotropic azoospermia and testicular adrenal rest tumors due to untreated simple virilizing congenital adrenal hyperplasia. Eur J Endocrinol.

[26] Sadeu JC, Hughes CL, Agarwal S, Foster WG (2010). Alcohol, drugs, caffeine, tobacco, and environmental contaminant exposure: reproductive health consequences and clinical implications. Crit Rev Toxicol.

[27] Dabaja AA, Schlegel PN (2014). Medical treatment of male infertility. Transl Androl Urol.

